# Towards a fair and transparent research participant compensation and reimbursement framework in Vietnam

**DOI:** 10.1093/inthealth/ihaa066

**Published:** 2020-11-09

**Authors:** Lucy J Sansom, Trang Pham Nguyen Minh, Iona E Hill, Quyen Nguyen Than Ha, Thuan Dang Trong, Celine Vidaillac, Nhu Dong Quynh, Hugo C Turner, Jennifer Ilo Van Nuil, Dung Nguyen Thi Phuong, Evelyne Kestelyn

**Affiliations:** Oxford University Clinical Research Unit, University of Oxford, Centre for Tropical Medicine, 764, Vo Van Kiet, Ho Chi Minh City, Vietnam; Oxford University Clinical Research Unit, University of Oxford, Centre for Tropical Medicine, 764, Vo Van Kiet, Ho Chi Minh City, Vietnam; Oxford University Clinical Research Unit, University of Oxford, Centre for Tropical Medicine, 764, Vo Van Kiet, Ho Chi Minh City, Vietnam; Department of Pure and Applied Chemistry, Technology and Innovation Centre, University of Strathclyde, 99 George St, Glasgow G1 1RD, UK; Oxford University Clinical Research Unit, University of Oxford, Centre for Tropical Medicine, 764, Vo Van Kiet, Ho Chi Minh City, Vietnam; Oxford University Clinical Research Unit, University of Oxford, Centre for Tropical Medicine, 764, Vo Van Kiet, Ho Chi Minh City, Vietnam; Oxford University Clinical Research Unit, University of Oxford, Centre for Tropical Medicine, 764, Vo Van Kiet, Ho Chi Minh City, Vietnam; Centre for Tropical Medicine and Global Health, New Richards Building, Old Road Campus, Roosevelt Drive, Oxford, OX3 7LG, UK; Oxford University Clinical Research Unit, University of Oxford, Centre for Tropical Medicine, 764, Vo Van Kiet, Ho Chi Minh City, Vietnam; Oxford University Clinical Research Unit, University of Oxford, Centre for Tropical Medicine, 764, Vo Van Kiet, Ho Chi Minh City, Vietnam; Centre for Tropical Medicine and Global Health, New Richards Building, Old Road Campus, Roosevelt Drive, Oxford, OX3 7LG, UK; MRC Centre for Global Infectious Disease Analysis, Department of Infectious Disease Epidemiology, School of Public Health, Faculty of Medicine, Imperial College London, Level 2, Faculty Building South Kensington Campus, London SW7 2AZ, UK; Oxford University Clinical Research Unit, University of Oxford, Centre for Tropical Medicine, 764, Vo Van Kiet, Ho Chi Minh City, Vietnam; Centre for Tropical Medicine and Global Health, New Richards Building, Old Road Campus, Roosevelt Drive, Oxford, OX3 7LG, UK; Oxford University Clinical Research Unit, University of Oxford, Centre for Tropical Medicine, 764, Vo Van Kiet, Ho Chi Minh City, Vietnam; Oxford University Clinical Research Unit, University of Oxford, Centre for Tropical Medicine, 764, Vo Van Kiet, Ho Chi Minh City, Vietnam; Centre for Tropical Medicine and Global Health, New Richards Building, Old Road Campus, Roosevelt Drive, Oxford, OX3 7LG, UK

**Keywords:** ethics, LMICs, participant compensation, participant reimbursement, remuneration framework, Vietnam

## Abstract

**Background:**

Providing compensation for participants in clinical research is well established and while international guidelines exist, defining a context-specific and fair compensation for participants in low-resource settings is challenging due to ethical concerns and the lack of practical, national compensation and reimbursement frameworks.

**Methods:**

We reviewed Oxford University Clinical Research Unit (OUCRU) internal reimbursement documentation over a 10-y period and conducted a scoping literature review to expand our knowledge of compensation and reimbursement practices including ethical concerns. We developed a preliminary reimbursement framework that was presented to community advisory boards (CAB) and clinical investigators to assess its applicability, fairness and transparency.

**Results:**

The main topics discussed at the workshops centered on fairness and whether the reimbursements could be perceived as financial incentives. Other decisive factors in the decision-making process were altruism and the loss of caregivers’ earnings. Investigators raised the issue of additional burdens, whereas the CAB members were focused on non-monetary elements such as the healthcare quality the patients would receive. All elements discussed were reviewed and, where possible, incorporated into the final framework.

**Conclusion:**

Our new reimbursement framework provides a consistent, fair and transparent decision-making process and will be implemented across all future OUCRU clinical research in Vietnam.

## Introduction

Providing compensation for participants in clinical research is a well-established practice, although defining a fair and appropriate amount remains a challenge and is still an area of debate.^1^^,^^2^ Researchers are guided by international guidelines such as the Council for International Organizations of Medical Sciences (CIOMS) statements and existing frameworks, but there is a need for enhanced transparency, greater consistency across research programs and practical models.^3^^–^^5^

For many years, discussions on the ethical issues surrounding participant payments have been ongoing, particularly related to a disproportionate research burden on vulnerable populations, coercion (pressure or intimidation) and undue influence, when an attractive offer affects a person's ability to make an informed decision.^6^^,^^7^ In low and middle income countries (LMICs), defining a context-specific and fair compensation for participants is challenging due to structural healthcare inequalities, pertinent ethical concerns and conflicting arguments about vulnerable populations, including aspects of economic disadvantages.^1^ Ethics committees have previously recommended that payments be kept low to avoid influencing participation by attracting participants through payment incentives.^8^^,^^9^ However, refraining from giving cash payments to poorer populations is not only unfairly discriminative, but this risks making samples within clinical research unrepresentative and it could be detrimental to recruitment if participants cannot afford to take part because their loss of earnings are not reimbursed.^2^^,^^10^

The Oxford University Clinical Research Unit (OUCRU) has conducted many research projects and clinical trials since its implementation in Vietnam in 1991 (http://www.oucru.org). Through a review of 134 studies conducted over the last 10 y, the clinical trials unit (CTU) reimbursement working group identified a lack of consistency and transparency in compensation of participants. Furthermore, OUCRU's reimbursement policy, last amended in 2013, sets a lump sum based on the participant's place of origin to cover a return journey using a public mode of transport, either bus or train. Due to the severity of participants’ conditions and occasionally cultural practices, participants often travel with relatives or caregivers, who frequently receive travel reimbursements as well. In absence of clear guidelines for reimbursements of non-participants, travel reimbursements cannot be accurately budgeted for, and this tends to be a contributor to overspent budgets in many studies.

Therefore, a standardized system is required to transparently and ethically justify how much participants in our studies should be reimbursed and why. The objectives of the present study are to develop a fair and transparent framework for research participant compensation, as well as to amend the current guidelines for travel reimbursement, with the hope this could help guide other research units in Vietnam and the wider region.

## Materials and Methods

### Document and literature review

The CTU reimbursement working group, consisting of eight people (three trialists, a medical anthropologist, a quality assurance manager, a contract manager and two study coordinators), reviewed the study protocols and patient information sheets of 134 clinical studies conducted at OUCRU from 2009 to 2019 to identify the current and past payment methods adopted by the unit for participants’ compensation and reimbursement (Supplementary Document 1). The working group agreed to use the following categories: (1) reimbursement of expenses incurred for trial visits, (2) compensation for participants’ time and burdens experienced in the trial and (3) incentive, either financial or a gift, usually intended to boost recruitment and retention.^5^^,^^11^ Building on in-house expertise as well as existing internal guidelines, a compensation framework from 2011 and the OUCRU reimbursement policy (Supplementary Figure 1), an initial list of key themes for compensation was identified: (1) the type of study (interventional or observational), (2) inpatient or outpatient study visits, (3) studies involving healthy volunteers, (4) hospitalization for study purposes, (5) risk, (6) invasiveness, (7) disease severity, (8) availability of treatment alternatives and (9) disease prevalence.

**Figure 1.**
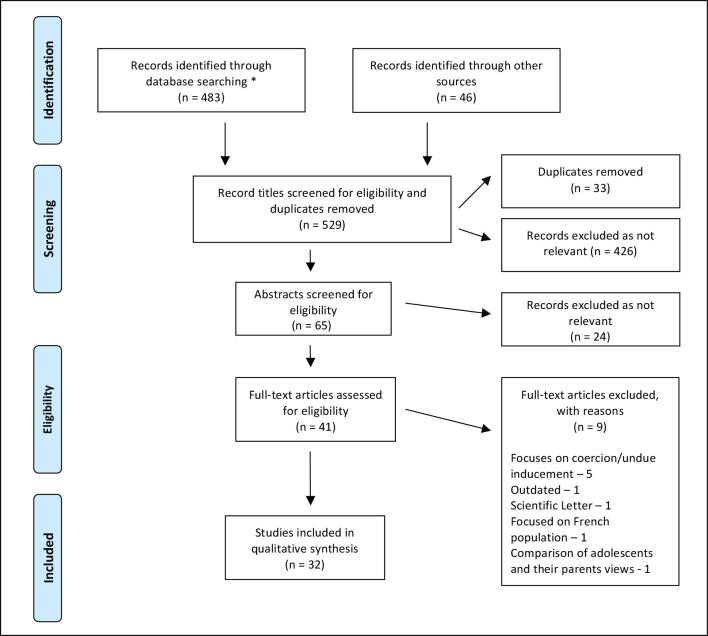
Diagram illustrating the scoping review results. ^*^PUBMED search (no date specified to ensure comprehensive overview of manuscripts) terms used in text (and/or) were: ‘reimbursement, participant, clinical trial, patient, compensation, ethics, research, low income’. Search terms used in title/abstract were: ‘reimbursement, payment, and remuneration’. Google Scholar search (2010+ to add only most recent manuscripts), which included the terms used in text: ‘patient compensation in clinical trials, participant reimbursement in clinical trials, participant reimbursement in clinical trials LMIC, research participation compensation low income setting, compensation reimbursement low resource participant’, as well as terms used in the title: ‘low income countries research, Vietnam healthcare’.

We then conducted a scoping literature review that took place from March to May 2019, aiming to further expand our knowledge on the themes identified and to explore the ethical concerns associated with participant compensation (Figure 1). We used the PubMed search tool (no date specified) as well as Google Scholar (manuscripts from 2010 onwards), only including original articles and reviews in English with a focus on LMICs. This yielded 483 journal articles. Forty-six records were also identified through reference lists and recommendations from colleagues. These included national and international compensation guidelines. We found many titles (n=426) to be irrelevant, focusing on compensation for injury, healthcare payment and hospital payment systems. After removing the duplicates (33 articles that appeared multiple times across the search terms), we screened 70 remaining titles and abstracts for eligibility. Subsequently, we reviewed 41 full-text papers, of which 32 were considered relevant in addressing the needs of our framework. Nine were not included; five of these because they focused on coercion and undue influence. Although briefly noted, these issues are beyond the scope of this paper.

### Compensation framework

Using the information gathered through the internal document review and from the literature, we re-examined the nine themes and their associated ethical concerns. Via an iterative process of in-house discussions within the CTU working group, we developed a points-based preliminary framework for compensation (Supplementary Figure 2). The value attributed for each point was determined using Oxford Tropical Research Ethics Committee and the Vietnamese Ministry of Health guidelines, which recommend that subjects should be compensated for loss of income, travel expenses and any other indirect losses.^12^^,^^13^ Furthermore, CIOMS suggests that the amount of compensation should be calculated using the minimum hourly wage in the region or country as a reference value.^3^ For Region 1, which includes Ho Chi Minh City and Hanoi, the minimum wage is 4420000 Vietnamese Dong (VND), which equals US$180 per month (https://wageindicator.org/salary/minimum-wage/vietnam). Considering that people in Vietnam work on average 25.5 days per month, this represents an approximate daily wage of 173 000 VND (approximately US$7.50). A value of 100 000 VND (approximately US$4) per point was considered appropriate as consistent with payments in OUCRU in the past. This preliminary framework focused on compensating participants for their loss of earnings and burdens endured, and deducting points if the participants had higher disease severity. To measure this objectively, we used the modified Rankin score (MRS), a validated measure of disability used across OUCRU studies. A higher MRS score equated to more points being deducted.

### Reimbursement element of the framework

Through review of OUCRU documentation and in-house discussion, it became apparent that reimbursement amounts were being paid to both participants and their family members/carers in many cases. This was not factored into the trial budget in the early stages and caused over-expenditure. Thus, we developed guidelines based on the participant's capability to travel alone. The MRS score was also utilized here as a method of determining who was eligible for additional reimbursement or not.

### Workshops

The preliminary points-based compensation and reimbursement framework was first presented in a workshop with patient representatives from community advisory boards (CAB) of two active trials (a hepatitis C trial, http://www.isrctn.com/ISRCTN17100273; and a TB meningitis trial, https://clinicaltrials.gov/ct2/show/NCT03100786? term = NCT03100786&rank = 1). Of the 12 attendees, 8 (67%) were involved in the aforementioned studies and 4 (33%) were relatives and had not been enrolled in any studies in the past. In Vietnamese, attendees were informed about the aims of the project, the ethical concerns surrounding patient reimbursement and the components of the framework. An interactive discussion was then conducted using a set of eight hypothetical scenarios based on the two current studies to compare current payment schedules with those generated by the framework (Supplementary Document 2). Using a questionnaire, we probed and collected attendees’ feedback on the payment amounts, factors considered in the framework, travel reimbursement guidelines and motivations to take part in clinical research (Supplementary Document 3). Another similar workshop was later presented in English to OUCRU staff. Ten attendees were present: clinicians, trialists, laboratory and public engagement managers, who all had first-hand experience in acting as principal investigators for clinical research. Feedback from the workshops led to a revised framework, which was then applied to 13 ongoing studies to compare the generated compensation amounts with those defined by the current policy (Supplementary Document 4). Applying the framework to these studies allowed us to evaluate its fitness for purpose and to address issues that remained unclear or were insufficiently defined. This led us to categorize types of burden more adequately and, additionally, we decided to increase the baseline pay for healthy volunteers before finalizing our compensation and reimbursement framework.

## RESULTS

**Figure 2.**
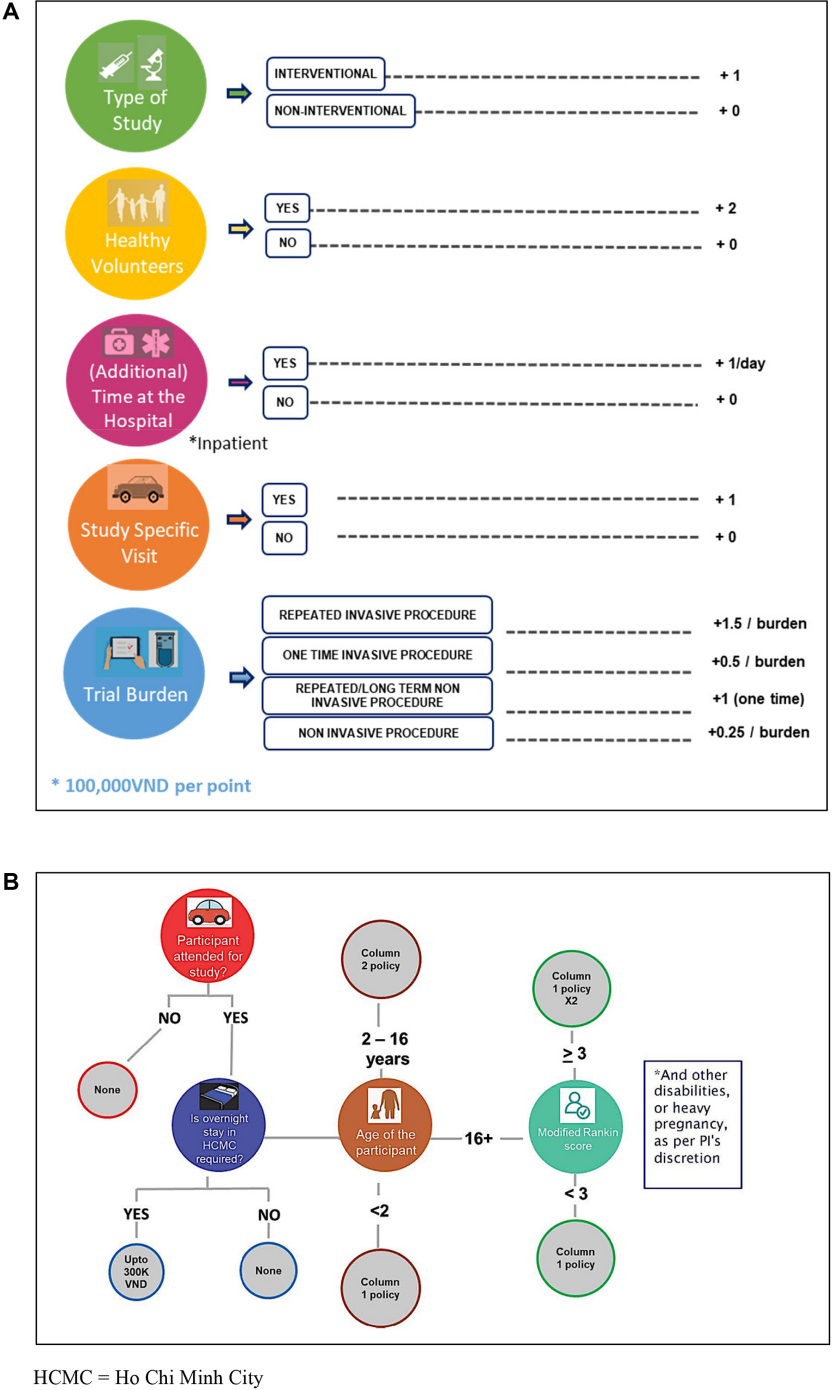
Final OUCRU compensation framework. (A) Point-based compensation framework designed to determine the amount participating patients are entitled to. The framework focuses on five key components: type of study, type of patient, hospital length of stay, study specific visit and burden. (B) Reimbursement section designed to determine whether reimbursement is given to patient and a family member/carer or an adult if the participant is a minor. HCMC, Ho Chi Minh City.

The final compensation and reimbursement framework results from a review of OUCRU internal guidelines, existing literature as well as the analysis of CAB and OUCRU investigators feedback, collected during two independent workshops (Figure 2).

### Compensation framework

The final compensation framework covers five central questions related to the design of the study and how it may affect participants’ wellbeing (Figure 2A). These questions are: (1) What type of study (interventional or observational) is conducted? (2) Is the participant a healthy volunteer, who enrolls in a clinical study with no prospect of medical benefit? (3) Is there any additional (not standard of care) inpatient hospital stay required per study protocol? (4) Are outpatient visits (not standard of care) required by the study protocol? (5) Are the participants subjected to burdens for study purposes only? Typical burdens were categorized as single- or long-term non-invasive (e.g. tracking devices, questionnaires, ultrasounds and x-rays) and single or repeated invasive procedures (e.g. blood samples, lumbar punctures and pharmacokinetic/pharmacodynamic sampling).

### Reimbursement element of the framework

The 2013 travel reimbursement policy will now be utilized alongside the following guidelines:

1. Participants are only entitled to travel reimbursement if they traveled to the study site, specifically and only for study purposes.
2. Participants with more severe disease or disability conditions (MRS≥3) and children (aged 2–16 y) are entitled to additional travel reimbursement to support the cost of caregivers and parents.

If participants are required to stay overnight, they are entitled to a maximum of 300 000 VND (approximately US$13) to account for hotel expenses.

### Key decisions and changes between the preliminary and final framework

First, following review of the current literature, it was apparent that clinical research should not compensate directly for risk related to a study procedure. Thus, we incorporated ‘additional burden’ as a factor into the preliminary framework presented at the workshops; one point for invasive procedures above that of standard care and one point for time-consuming or burdensome tasks, such as questionnaires. However, this proved contentious. CAB members stated that they did not fully comprehend what constitutes additional burden, such as additional blood volume or tests above that of routine care. OUCRU investigators stated that studies conducted at OUCRU vary significantly in the number of study visits and the number and intensity of study procedures. They felt that the framework presented would not differentiate one study with many non-invasive but time-consuming tasks from another involving a small number of highly invasive procedures. All clinicians agreed that participants should be compensated for each burden encountered above that of standard of care. Following this, typical ‘burdens’ across all OUCRU research studies were categorised into non-invasive (single- or repeated/long-term) or invasive (single or repeated) procedures.

Second, the preliminary framework originally incorporated minus points into the algorithm to compensate for higher hospital costs associated with more severe levels of disease, and the hypothesis that these patients are unlikely to be working and therefore should not be compensated for loss of earnings. We therefore incorporated the MRS in our framework. Participants with a higher MRS score had more points deducted (Supplementary Figure 2). This resulted in different payments for patients enrolled in the same study, as shown in the scenarios 1 vs 2 (Supplementary Document 2). The CAB members struggled to understand the algorithm and half of the attendees disagreed that patients of differing disease severity should receive less or different financial compensation in the hypothetical scenarios. They felt that participants should either get equal compensation, regardless of the severity of their disease, or, if anything, that people with more severe disease should receive more compensation, as they have to stay longer in hospital. OUCRU investigators agreed with the algorithm but felt that it could be difficult to explain to two patients in the same study, who might be next to each other on the ward or in the outpatient clinic, why one person received more compensation than the other. The OUCRU investigators also agreed that degree of disease severity should not be a factor in financial compensation. As a result, disease severity was removed from the framework and payment was made consistent across all participant arms.

Third, the preliminary framework awarded an additional point if the safety profile of the intervention was not well known. This raised questions in the OUCRU investigator workshop as some interventions’ safety profiles are well known but not in the context in which they are to be used. Additionally, the original 2011 OUCRU guidelines show higher payments for interventional studies. Therefore, one point was awarded at baseline for interventional trials in the final framework instead.

Last, the preliminary framework included a multiplication factor for interventional studies (X2). This additional step proved to be an unnecessary complication and, instead, the final framework has a point value of 100000 VND (approximately US$4) rather than 50000 VND (approximately US$2) and a baseline payment for interventional studies (as discussed).

### General feedback on participant compensation amounts

After discussion of the preliminary framework and working through the different scenarios, the CAB members felt that the proposed compensation amounts were fair in most of the scenarios. Interestingly, one person felt that using the minimum wage as a guide for payments was too low and suggested that salary scales for government employees should be used. Another patient representative felt that the compensation amount was too low to cover costs for each follow-up visit. Consistent with this, OUCRU investigators said that follow-up visit payments of 50 000 VND (approximately US$2) were too low for the participants’ time but agreed that it is an appropriate gesture as most OUCRU studies currently do not give any compensation on top of travel reimbursement. One investigator, however, commented that they have experienced loss to follow-up as relatives refuse to accompany the participant on follow-up visits. Although travel reimbursement is offered for both the participant and the carer, the carer does not want to lose a day's wages. Notably, and of great importance, the CAB members confirmed that in the context of the tested scenarios, the financial factor did not represent the primary motivation. They stated that their main motivation to participate would be to learn more about the disease they had and to help others in the community.

### Travel reimbursement

Workshop attendees were overall satisfied with the proposed reimbursement guidelines. One CAB member suggested that travel time should be included in the reimbursement value. This suggestion was debated but, given that the amount reimbursed to participants is higher than the actual cost of public transport to a hospital in Ho Chi Minh City, the CTU working group felt this covered both costs as well as travel time and hence this was not included in the final framework. Additionally, the OUCRU investigators agreed that certain conditions, such as pregnancy or limited vision, may not fall into the predefined MRS, but still require a relative to accompany the participant. In such situations, the principal investigator has the flexibility to make the final decision regarding the travel reimbursement amount.

## Discussion

A consensus in the literature is that reimbursement for incurred costs such as transport, subsistence and accommodation as well as compensation for time and burden is acceptable, and often encouraged, as long as it is not so high as to coerce a participant.^2^^,^^4^^–^^6^^,^^14^ The final framework covers both travel reimbursement and participant compensation as agreed upon by the CAB and the OUCRU investigators; however, several remuneration models incorporate an element of incentive to participate or a form of appreciation.^1^^,^^2^^,^^4^^,^^5^ While the working group acknowledges that financial incentives can send participants a strong message that researchers value their time and increases their willingness to participate, the concern of ‘undue inducement’ is present and particularly heightened in the context of structural inequalities existing in LMICs.^8^^,^^15^^–^^17^ On the other hand, stopping participants from receiving compensation in LMICs can act as a hindrance to study recruitment, as studies conducted in Kenya showed that low payments to participants from families reliant on subsistence forms of livelihood can impact the ability of breadwinners to feed their families.^11^^,^^18^

After discussing whether to add this element to the framework, the working group opted not to include financial incentives. In addition to a limited level of ‘recruitment competition’, the inducement issues mentioned above are further complicated by contexts with structural inequalities, where access to healthcare is limited and decisions are less driven by individual choice or money, and more by the prospect of healthcare. Studies have shown that access to high-quality treatment, which is otherwise unaffordable or unavailable, is a contributing factor to participating in research in LMIC settings, despite the possibility of receiving a placebo or unknown side effects.^19^^–^^21^ Consequently, this raises concerns that attractive medical benefits can cause coercion because participants have no alternative choice or are perceived as ‘an empty choice’, as first described by Kingori.^22^ We felt this was less of an issue in Vietnam, where healthcare is available and a health insurance scheme was introduced in 1993, which aimed for a target of 90% coverage by 2020.^23^ In addition to monetary compensation and healthcare benefits, wanting to contribute to research was often mentioned.^24^ Results from the CAB workshop support the studies conducted by Njue et al. and Shah et al., who reported that patient representatives agreed that participants could not be financially persuaded to take part and that they wanted to contribute to research to help people in their communities.^24^^,^^25^

Throughout the development of the framework, the working group wanted to consider other factors that could influence a participant's willingness or motivation to participate. We did not want to lose sight of the importance of our local setting and cultural aspects that could impact participant remuneration procedures. Studies in Kenya and Gambia saw financial benefits as problematic; for example, men felt suspicious about receiving cash to compensate for blood samples and this made them wonder about the real use of their blood.^11^^,^^18^^,^^26^ Similarly, women felt uncomfortable with the compensation received for their participation, knowing it would likely result in conflict with their spouses.^11^ Although suspicions surrounding financial compensation do exist in Vietnam, this seems to be perceived as more problematic by clinicians and less so by participants. Vietnam is also more liberal in terms of gender hierarchy, however, in some Asian cultures, it is unheard of, or even frowned upon, for a woman to travel without her partner.^27^^,^^28^ For the working group it was important to consider the distance that participants may be required to travel, and older generations (women in particular) might not feel comfortable traveling alone. The OUCRU travel policy reimburses a set amount according to the province where the participant lives/is traveling from (i.e. the participant is not required to retain receipts for reimbursement). This allows for flexibility in that they can use that money to travel via the mode that suits them, for example, by car or motorbike, or with a relative if they prefer to have company.

In addition to unpacking the content and meaning of burden and disease severity for our remuneration framework, we reviewed other elements in the research process such as a complex informed consent process. This is beyond the scope of this paper and is being explored in other OUCRU research projects.^28^ Finally, we wanted to highlight the inclusion of ‘healthy volunteers’ as an element in the framework. These volunteers are exposed to the risk and discomfort of research without the potential or expectation of health benefits. Since there is no medical incentive for the participant often the primary motivation seems to be financial reward but again the literature shows that in this context altruism and contributing to research and knowledge are important motivators.^25^^,^^29^^–^^33^ We intend to apply this framework to planned human challenge studies involving healthy participants and, if appropriate, revise and adapt our current framework.

### Conclusions

The framework provides a transparent, fair and consistent method of calculating compensation for research participants by itemizing factors that contribute to the total amount. This tool can be used across all OUCRU clinical research and is a means of providing justification for the payment to local and international ethics committees. It is not only practical in its use but is also underpinned by sound ethical considerations with acknowledgment of elements important to study teams and participants such as additional burdens and disease severity. Engaging the CAB and the OUCRU investigators in the development process of this framework was extremely valuable and helped the reimbursement working group to gain understanding of and insight into the lived experiences of participants and study teams with regard to research participation and compensation.

This framework can be applied to other clinical trials units in Vietnam and our OUCRU units in Indonesia and Nepal. Currency values to substitute points should stay in line with CIOMS guidelines that state compensation should reflect the minimum wage in a particular country.

## Supplementary data

Supplementary data are available at *International Health* online.

## Supplementary Material

ihaa066_Supplemental_File
